# Treatment Costs of Pneumonia, Meningitis, Sepsis, and Other Diseases among Hospitalized Children in Viet Nam

**DOI:** 10.3329/jhpn.v28i5.6151

**Published:** 2010-10

**Authors:** Dang Duc Anh, Arthorn Riewpaiboon, Le Huu Tho, Soon Ae Kim, Batmunkh Nyambat, Paul Kilgore

**Affiliations:** ^1^ National Institute of Hygiene and Epidemiology, Hanoi, Viet Nam; ^2^ Division of Social and Administrative Pharmacy, Faculty of Pharmacy, Mahidol University, Bangkok, Thailand; ^3^ Khanh Hoa Provincial Public Health Service, Nha Trang, Viet Nam; ^4^ International Vaccine Institute, Seoul, South Korea

**Keywords:** Cost-of-illness, Healthcare costs, Meningitis, Pneumococcal diseases, Pneumonia, Sepsis, Viet Nam

## Abstract

The aim of this study was to estimate the costs of treatment of children who present with the signs and symptoms of invasive bacterial diseases in Khanh Hoa province, Viet Nam. The study was an incidence-based cost-of-illness analysis from the health system perspective. The hospital costs included labour, materials and capital costs, both direct and indirect. Costs were determined for 980 children, with an average age of 12.67 months (standard deviation±11.38), who were enrolled in a prospective surveillance at the Khanh Hoa General Hospital during 2005-2006. Of them, 57% were male. By disease-category, 80% were suspected of having pneumonia, 8% meningitis, 3% very severe disease consistent with pneumococcal sepsis, and 9% other diseases. Treatment costs for suspected pneumonia, meningitis, very severe disease, and other diseases were US$ 31, US$ 57, US$ 73, and US$ 24 respectively. Costs ranged from US$ 24 to US$ 164 across different case-categories. Both type of disease and age of patient had statistically significant effects on treatment costs. The results showed that treatment costs for bacterial diseases in children were considerable and might differ by as much as seven times among invasive pneumococcal diseases. Changes in costs were sensitive to both age of patient and case-category. These cost-of-illness data will be an important component in the overall evidence base to guide the development of vaccine policy in Viet Nam.

## INTRODUCTION

Globally, nearly two million children die each year due to acute respiratory tract infections (ARIs) (1). Besides, an estimated 150 million episodes of childhood pneumonia occur, of which 11-20 million require hospitalization (2). The incidence of pneumonia among children aged less than five years is estimated to be 0.29 episodes per child-year in developing and 0.05 episodes per child-year in developed countries (2). Recent estimates from the United Nations Children's Fund suggest that *Streptococcus pneumoniae* causes half of all deaths due to pneumonia in children, and 70% of these deaths occur in Africa and Asia (3). A large proportion of these deaths could be averted through routine immunization using available pneumococcal conjugate vaccines (4,5). However, a substantial barrier to the use of pneumococcal conjugate vaccine is the non-availability of adequate information on costs associated with treatment of clinical conditions consistent with invasive pneumococcal diseases in children. In addition, an increasing number of countries are now requesting or requiring cost-of-illness data on vaccine-preventable diseases before making long-term commitments for the introduction of new vaccines. At present, only a limited number of studies reported costs associated with syndromes of invasive bacterial infections in children from developing countries (6–8). To address this gap in information, we determined costs for the treatment of invasive bacterial infection syndromes in children in Nha Trang, Viet Nam.

## MATERIALS AND METHODS

### Study design and data collection

We conducted an incidence-based cost-of-illness analysis from the healthcare system perspective, a technique used for measuring the economic burden of patients from the onset to the end of illness (9,10). We selected the Khanh Hoa General Hospital (KHGH) for this study in Nha Trang city, the capital of Khanh Hoa province, Viet Nam. During 2005-2006, a prospective surveillance for clinical syndromes associated with invasive pneumococcal diseases was conducted at the KHGH; its results were published elsewhere (11). Before the enrollment of study patients, all clinicians and medical staff of the Department of Pediatrics and Infectious Diseases of KHGH were provided with standardized procedures and screening criteria for identifying children with pneumonia, meningitis, sepsis, and other syndromes consistent with invasive bacterial diseases. After the completion of informed consent procedures, appropriate clinical specimens (blood and/or cerebrospinal fluid) were obtained, and parents were interviewed to collect demographic information and illness history. All specimens were tested using bacterial culture methods, and selected specimens underwent further testing by antigen detection and polymerase chain reaction. Definitions of diseases were based on the published guidelines (12–13), and patients’ demographics and clinical characteristics were drawn from the published research report (11).

### Costing methods

For our analysis, cost-of-illness was defined as the sum of direct medical, direct non-medical, and indirect costs (9). Direct medical cost was defined as costs directly associated with provision of healthcare, including costs for detection, treatment, continuing care, rehabilitation, and terminal care. For this study, we focused our analysis on direct medical or treatment costs employing the micro-costing approach that estimates costs of treatment by summing all medical services received by an individual patient (14). Vietnamese children, aged less than six years, are now covered under a national health insurance supported by the Vietnamese government. In Viet Nam, public hospitals receive government funding in the form of lump sum budget and insurance system reimbursements for delivery of medical services to patients. Hospitals receive revenue in the form of out-of-pocket payments directly from patients (approximately 60% of the total expenditure). For the health insurance, hospital costs are reimbursed by the Ministry of Health using a fee-for-service system. To collect data on individual patient's cost, the KHGH uses a FoxPro® (Microsoft, Redmond, Washington, DC) data-entry system that records costs of services provided (e.g. number of hospitalization days, laboratory tests, radiographic procedures, therapeutic procedures, and drugs) to an individual patient.

To compute costs of each medical service, we used the ratio of costs to charges (RCCs) method (15,16). In this method, the RCC takes into account all costs incurred by the hospital and the hospital's total revenue (i.e. patient-charge in the form of out-of-pocket payment from uninsured patients and reimbursement from the Ministry of Health for insured patients). Then, the total annual hospital costs were divided by total annual revenue (or total charges) to derive the overall RCC. We then multiplied charges for each service by the RCC to obtain costs of a service item for each patient. We summed the costs of all medical services and drugs of each patient to obtain total direct medical costs or treatment costs for each patient. Total hospital cost included labour cost of all hospital staff, material cost (e.g. use of electrical utilities), and capital cost. Capital costs associated with the use of depreciable assets, i.e. equipment, buildings, furnishings, and vehicles. The capital cost was calculated based on the straight-line equivalent annual economics-based approach (17). This approach covers both depreciation cost (the rate at which the capital asset is ‘used up’) and opportunity cost (interest) of making the investment (18). The cost is equally allocated to period of useful life. Based on the Ministry of Finance guidelines, we assumed that the useful life of vehicles, equipment, and buildings was 5, 10 and 25 years respectively (19). We covered all items in use, although they were over official working years since they still had opportunity cost (18). We applied a 3% discount rate to our cost analysis (18). The costs were converted from Viet Nam dong to US dollar at the 2006 prices (VND 15,976.83=US$ 1) (20).

### Analysis of data

Demographic information was described by frequency and proportion. We calculated means with standard deviations (SDs) for continuous variables and used multiple regression analysis to explore the factors associated with variations in treatment costs. Covariates considered in this modelling included age (months), duration of stay (days), and diagnosis. In addition, since prices of drugs may vary substantially among hospitals, we performed a sensitivity analysis to explore the effect of variation in prices of antibiotics across a range of prices of antibiotics available from the KHGH databases.

## RESULTS

### Characteristics of patients

Patients’ demographics are presented in Table 1. In total, 980 children were enrolled in the study. Their average age was 12.67 months (SD±11.38). The majority (56.5%) were male. Sixty percent of the patients were currently breastfeeding, and 42% had received antibiotics during the 14-day period before hospital admission. Of the 980 children, 80.4% were diagnosed with pneumonia, 7.8% with meningitis, 3.2% with very severe disease, and 8.7% with other diseases. Of the 980 children with a specific diagnosis, 43.5% were classified as CXR-confirmed pneumonia, followed by CXR-confirmed severe pneumonia (19.7%) and suspected meningitis (6.7%). All the enrolled patients were treated in the inpatient facilities of the KHGH, and the average duration of hospitalization was 6.48 days (SD±4.14) (Table 2).

**Table 1.** Demographic characteristics of study patients (n=980)

Characteristics	No.	%
Age-group (months)		
≤11	521	53.2
12-23	290	29.6
24-35	116	11.8
36-47	33	3.4
48-59	20	2.0
Gender		
Male	554	56.5
Female	426	43.5

**Table 2.** Distribution of patients, duration of stay, and treatment cost by case-category

Diagnosis	Patients		Length of stay, mean days (±SD)	Cost (US$) (±SD)			
	No.	%		Drug	Laboratory	Hotel*	Total
All pneumonia	788	80.4	6.54 (4.26)	15.29 (26.77)	4.09 (7.8)	11.42 (7.82)	8.8 (35.76)
Probable pneumonia	40	4.1	7.15 (4.98)	12.61 (15.2)	3.6 (4.38)	11.28 (8.16)	27.48 (25.84)
CXR-confirmed pneumonia**	426	43.5	6.72 (3.78)	10.22 (12.75)	3.51 (7.12)	11.03 (6.64)	24.75 (20.74)
Probable severe pneumonia	59	6.0	6.21 (3.27)	17.55 (29.43)	4.64 (6.18)	10.27 (6.37)	32.45 (36.58)
CXR-confirmed severe pneumonia**	193	19.7	6.35 (2.70)	17.65 (33.97)	3.93 (6.03)	12.56 (9.99)	34.14 (45.21)
Probable very severe pneumonia	26	2.7	6.35 (2.70)	48.53 (53.09)	9.37 (16.74)	11.15 (9.64)	69.04 (65.82)
CXR-confirmed very severe pneumonia**	44	4.5	5.82 (2.98)	33.82 (44.98)	7.03 (13.74)	12.1 (7.58)	52.95 (57.25)
All meningitis	76	7.8	6.42 (4.61)	29.11 (42.3)	10.93 (19.49)	16.57 (11.23)	56.61 (61.4)
Suspected meningitis	66	6.7	6.64 (4.61)	20.03 (29.05)	8.82 (16.72)	14.75 (10.08)	43.61 (41.38)
Probable bacterial meningitis	5	0.5	6.60 (5.55)	71.86 (36.26)	19.62 (5.44)	29.41 (16.61)	120.89 (52.45)
Definite meningitis	5	0.5	3.40 (3.05)	106.16 (86.41)	30.1 (43.84)	27.7 (4.8)	163.95 (133.22)
Very severe disease	31	3.2	6.71 (3.31)	46.2 (73.1)	10.2 (22.85)	17.09 (17.43)	73.48 (106.11)
Other	85	8.7	5.95 (2.64)	9.53 (17.34)	3.77 (5.6)	10.62 (7.45)	23.92 (25.95)
Total	980	100	6.48 (4.14)	11.64 (30.79)	4.79 (10.05)	11.93 (8.70)	33.56 (42.72)

*Hotel cost denotes costs of room, meal, and routine nursing care in inpatient ward;

**CXR denotes chest radiograph; SD=Standard deviation

### Estimation of costs

To convert the payment to economic cost, the cost-to-charge ratio method was applied. The KHGH had total costs of VND 172,482 million with revenue of VND 151,219 million or a hospital cost-to-charge ratio of 1.14. Of 48 antibiotics used for treating the study patients, ceftriaxone, cefotaxime, and cefuroxime were the most expensive drugs (Table 3). These three antibiotics accounted for 38%, 29%, and 16% of all the drug costs in the study respectively. The average treatment or direct medical costs of pneumonia, meningitis, very severe diseases, and other diseases were US$ 31, US$ 57, US$ 73, and US$ 24 respectively (Table 2). The cost of drugs represented 40-63% of the total treatment costs. The highest costs (mean US$ 164) was incurred for definite (i.e. laboratory-confirmed) meningitis, followed by probable bacterial meningitis (US$ 121). Results of sensitivity analysis showed that treatment costs rose by 8% and decreased by 4% when the maximum and minimum prices of antibiotics were used for analysis instead of mean prices respectively. The costs increased by 13% when calculated using maximum compared to minimum prices of antibiotics.

**Table 3.** Prices of selected antibiotics and total cost of antibiotic use by patients (n=980)

Antibiotic	Vial quantity	Average unit price (US$)	Total cost (US$)	% of total
Ceftriaxone	250 mg	4.24	3,557.82	38.4
Cefotaxime	1 g	1.35	2,704.71	29.2
Cefuroxime	250 mg	0.46	1,469.46	15.8
Ciprofloxacin	200 mg	12.44	181.22	2.0
Others	NA	NA	1,364.52	14.6

NA=Not applicable

To explore the co-factors affecting the treatment costs, we conducted a multiple regression analysis using age, days of hospitalization, and diagnosis as independent variables. All, except days of hospitalization, had significant effects on cost. The model had explanation power of 17.2% (adjusted R^2^=0.172). Estimated costs regarding age and diagnosis were constructed. The costs of all disease-groups were inversely related to age—as the age of patients increased, costs decreased (Fig.). When we compared diagnosis and age-groups, the average treatment cost of pneumonia ranged from US$ 18 to US$ 66. Similarly, the cost of treating meningitis ranged from US$ 25 to US$ 190.

**Fig.**
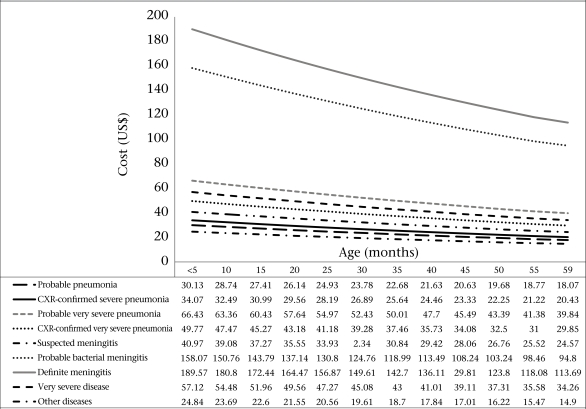
Age-specific treatment cost estimated by modelling

CXR denotes chest radiograph

## DISCUSSION

### Characteristics of patients

This study used data on patients from a representative group of hospitalized children in Viet Nam. As in previous studies (21,22), most (94.6%) children hospitalized with acute respiratory disease were aged less than three years. Analysis of data from the prospective surveillance suggests that most (80.4%) suspected invasive pneumococcal diseases in Vietnamese children manifests initially as pneumonia. While a few comparative studies across developing countries have been performed, data from our surveillance study in Khanh Hoa province suggest that children suffered from various acute lower respiratory tract diseases due to respiratory bacteria and viruses. While some children with bacterial infections may present with less severe disease due to prior outpatient treatment with antibiotics, such empiric treatment may put the child at risk for prolonged disease with antibiotic-resistant bacteria or respiratory viruses that do not respond to treatment with antibiotics (23).

### Costing of illness

Two previous studies in Pakistan estimated costs associated with clinical syndromes of invasive pneumococcal disease (24,25). For hospitalized care, the healthcare provider spent, on average, US$ 71 per episode for pneumonia, US$ 235 for severe pneumonia, and US$ 2,043 for meningitis (25). Results of another study showed that the average treatment cost per episode was US$ 17.77 for pneumonia and US$ 125.29 for severe pneumonia (24). However, direct comparison of healthcare costs across countries is limited by the fact that these costs arise from systems with different approaches to healthcare delivery, treatment patterns, drug-pricing, costing of services, and different time periods for data analysis. Nevertheless, a comparison of trends in costs over time and the proportionate distribution of costs are reasonable. As in our analysis, data from Pakistan showed that the cost of treatment of meningitis was higher than that of pneumonia, and cost of treatment of severe pneumonia was higher than that of non-severe pneumonia. Our data in Viet Nam also showed that the cost of treatment of meningitis was approximately twice compared to that of pneumonia. In contrast, this difference in the cost of treatment of meningitis compared to that of pneumonia was nearly 30-fold in Pakistan. In the subset of patients with laboratory-confirmed disease, the cost of treatment of definite meningitis (US$ 163.95) was five times higher than that of CXR-confirmed pneumonia (US$ 32.45). Another method of comparison is to compare with the country's economy indicators. The average treatment cost (US$ 33.56) was approximately one-tenth of the Viet Nam per-capita gross national product (GNP). The per-capita 1998 GNP for Viet Nam was US$ 350 (26).

Because of limitations in the availability of data, we could not determine an accurate count of material consumption. Instead, we assumed that quantities of materials used were equivalent to the amount of materials purchased. Regarding the cost-to-charge method applied in this study, the accuracy decreased if some patient-services were provided without charge as total hospital costs covered all costs of services produced. Thus, the cost of free services, e.g. health education, would be absorbed by services for which charges were levied. Accuracy was also related to accuracy of price-setting (27). This refers to the relative prices of different services (compared to each other in the same hospital) actually reflects the relative resource-use of those services, even if the level of those prices is much higher than the cost.

We considered resource-consumption patterns and the representativeness of the study hospital compared to other hospitals in Viet Nam. In the study hospital, costs of materials (54%), labour (28%), and capital (18%) were all similar to those of Thailand (27–29). For the cost-to-charge ratio, a 2003 study in the same hospital found that the ratios for the laboratory, imaging diagnosis, and the whole hospital were 1.45, 0.74, and 0.98 respectively (30). The difference might be due to different methods used for determining both costs and hospital revenues.

### Factors affecting treatment costs

In general, consumption of drugs was positively associated with both body-weight and age. Due to the relatively low explanatory power of the fitted cost model (adjusted R^2^=0.172), other variables not included in our modelling procedures may explain variations in cost. In addition, the relationship of the independent variables may be confounded by age. For example, younger children may be more vulnerable to pneumococcal diseases than older children. In addition, drug-prescribing behaviours may affect both use of certain drugs and costs of drugs provided (31). We were unable to include factors relating to the attending physician in the model tested.

### Generalizability

We believe that data used in our analysis are both reliable and internally valid. The KHGH has a well-organized computerized database, from which all costing data were drawn. Our findings regarding age- and disease-specific treatment costs can be used together with age-specific incidence rates for such economic evaluations as cost-effectiveness, cost-utility, and cost-benefit analyses (32). Age-specific data are also useful for assessing the most cost-effective ages for immunizations.

### Conclusions

Treatment cost of pneumococcal diseases was considerable in Viet Nam. Costs of suspected/probable and confirmed pneumococcal disease were statistically different as were costs of pneumococcal pneumonia and meningitis, which can differ by as much as seven times. Age of patient and type of disease affect costs of treating pneumococcal diseases in Viet Nam. These data will be important in the overall evidence to guide the development of vaccine policy in Viet Nam.

## ACKNOWLEDGEMENTS

The study was supported by the PneumoADIP and by the Governments of Kuwait, Sweden, and South Korea.

The authors thank Dr. Le Thi Phuong, Mr. Nguyen Thanh Hien, Mr. Nguyen Vu Cuong, and Mr. Van Hoi for superb work in data collection and assistance for data management and Kathy Murray for editorial assistance.
